# ParPEST: a pipeline for EST data analysis based on parallel computing

**DOI:** 10.1186/1471-2105-6-S4-S9

**Published:** 2005-12-01

**Authors:** Nunzio D'Agostino, Mario Aversano, Maria Luisa Chiusano

**Affiliations:** 1 Department of Structural and Functional Biology, University 'Federico II', 80134 Naples, Italy

## Abstract

**Background:**

Expressed Sequence Tags (ESTs) are short and error-prone DNA sequences generated from the 5' and 3' ends of randomly selected cDNA clones. They provide an important resource for comparative and functional genomic studies and, moreover, represent a reliable information for the annotation of genomic sequences. Because of the advances in biotechnologies, ESTs are daily determined in the form of large datasets. Therefore, suitable and efficient bioinformatic approaches are necessary to organize data related information content for further investigations.

**Results:**

We implemented ParPEST (**Par**allel **P**rocessing of **EST**s), a pipeline based on parallel computing for EST analysis. The results are organized in a suitable data warehouse to provide a starting point to mine expressed sequence datasets. The collected information is useful for investigations on data quality and on data information content, enriched also by a preliminary functional annotation.

**Conclusion:**

The pipeline presented here has been developed to perform an exhaustive and reliable analysis on EST data and to provide a curated set of information based on a relational database. Moreover, it is designed to reduce execution time of the specific steps required for a complete analysis using distributed processes and parallelized software. It is conceived to run on low requiring hardware components, to fulfill increasing demand, typical of the data used, and scalability at affordable costs.

## Background

The role of bioinformatics to support the Life Sciences has become fundamental for the collection, the management and the interpretation of large amount of biological data. The data are in most cases derived from experimental methodologies with large scale approaches, the so-called "omics" projects. International projects aimed to sequence the whole genomes of model organisms are often paralleled by initiatives for the expressed data sequencing to support gene identification and functional characterizations. Moreover, because of advances in biotechnologies, ESTs are daily determined in the form of large datasets from many different laboratories. Therefore, the analyses of expressed sequence data involve the necessity of suitable and efficient methodologies to provide high quality information for further investigations. Furthermore, suitable models for the organization of information related to EST data collections are fundamental to provide a preliminary environment for analyses of structural features of the data, as well as of expression maps and of functional relationships useful for the interpretation of mechanisms and of rules of gene expression processes.

There are many software available for EST processing, with the purpose to clean the datasets from contaminations [1-4] and to cluster sequences sharing identities to assemble contigs [5-10]. Sequences cleaned from contaminations are usually submitted to the dbEST database as they represent a fundamental source of information for the scientific community [11-13]. The results of the clustering step are useful to analyse sequence redundancy and variants as they could represent products of the same gene or of gene families. Moreover, ESTs or contigs obtained from the clustering step are usually analysed by comparisons with biological databanks to provide preliminary functional annotations [14]. On the other hand, few efforts are known where all the sets of consecutive steps for EST processing, clustering and annotation are integrated into a single procedure [15-17].

Expressed sequence curated databanks are worldwide available. They consist of collections built starting from dbEST, using selected computational tools to solve the complex series of consecutive analyses. Some of the well known efforts are the Unigene database [18,19], the TIGR gene indices [20] and the STACK project [21,22].

Our contribution to this research is a pipeline, named ParPEST (**Par**allel **P**rocessing of **EST**s), for the pre-processing, clustering, assembling and preliminary annotation of ESTs, based on parallel computing and on automatic information storage. Useful information resulting from each single step of the pipeline is integrated into a relational database and can be analysed by Structured Query Language (SQL) calls for a "ad hoc" data-mining. We also provide a web interface with suitable pre-defined queries to the database for interactive browsing of the results that is supported by graphical views.

## Methods

**Figure 1:**
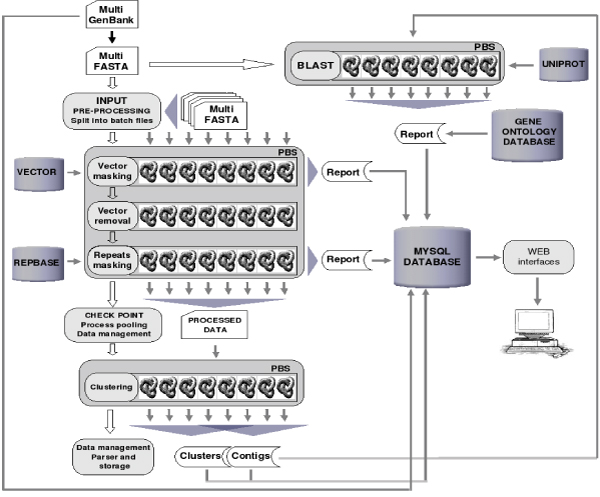
**Schematic view of ParPEST pipeline**. EST sequences in GenBank or FASTA format can be submitted to the pipeline. ParPEST performs automatically the consecutive processes (ESTS cleaning, clustering, assembling and BLAST comparisons) as represented by blank arrows (⇒). Data flow is represented by simple arrows (→). Databases supporting the analysis are included. The results are adequately reported and organized into a MySQL relational database indicated too. As reported, the database can be queried by SQL calls and is accessible to users by web based intuitive interfaces.

The inputs to ParPEST can be raw EST data provided as multi-FASTA files or in GenBank format. The pipeline allows pre-processing, clustering and assembling of ESTs into contigs and functional annotation of both raw EST data and resulting contigs (Figure 1) using parallel computing.

The pipeline has been implemented using public software integrated by in-house developed Perl scripts, on a 'Beowulf class' cluster, with Linux (Red Hat Fedora Core 2) as default operating system and the OSCAR 4.0 distribution [23] that provides the tools and the software packages for cluster management and parallel job executions.

The main process of the pipeline is designed to serialize and to control the parallel execution of the different steps required for the analysis and to parse into reports the collected results.

Input datasets are parsed by a specific routine so that information from the GenBank format or included in the FASTA format could be upload into a MySQL database.

Sequence data are pre-processed in two steps, to clean the data and to avoid mis-clustering and/or mis-assembling. The first step requires RepeatMasker [4] and the NCBI's VECTOR database [24] for checking vector contaminations. In the second step, RepeatMasker and RepBase [25] are used for filtering and masking low complexity sub-sequences and interspersed repeats. To accomplish sequence pre-processing a specific utility has been designed to distribute the tasks across the computing nodes. Job assignments are managed by a PBS batch system [23]. Job control at each step and output files integration is managed by the main process.

PaCE [6] is the software we selected for the clustering step. For a parallel execution it requires an MPI implementation and a job scheduler server. Once the whole pre-processed sequences are clustered, they are assembled into contigs using CAP3 [26]. To exploit the efficiency of CAP3 and to avoid the overhead time consuming of PBS, the main process we implemented has been designed to bundle groups of commands to be executed sequentially by each processor.

The functional annotation is performed using the MPI-Blast package [27]. Raw EST data and assembled contigs are compared using BLASTx versus UNIPROT database [28]. The blast search is performed setting an E-value less equal than 1. In case of successful matches, the five best hits are reported. When the subject accession number is reported in the Gene Ontology (GO) database [29,30] the corresponding classification is included to further describe the putative functionalities. Moreover, links to the KEGG database [31] are provided via the ENZYME [32] identifier in the resulting report, for investigations on metabolic pathways.

All the results obtained from the single steps of the pipeline are recorded in a relational database and are managed through SQL calls implemented in a suitable PHP-based web interface to allow interactive browsing of all the structural features of each EST, their organization in the assembled contigs, the BLAST-derived annotations as well as the GO classifications.

## Results and discussion

### Efficiency

The pipeline performs a parallel analysis on large amount of EST data. Because of distributed computing there is no execution limit for the processes, that are allocated according to available resources. The free release of PaCE, [6] that we experienced to be limited at 30.000 sequences, has been updated with the latest version provided by the authors who successfully tested the software with more than 200.000 sequences (personnel communication). Therefore, the only limiting factor for the complete execution of the pipeline is the memory space required for the database storage.

The pipeline has been tested on a cluster of 8 nodes single processor. In Table 1, execution times (in seconds) are reported for 5 different dataset sizes (randomly selected ESTs) and for different node configurations (4, 6 and 8 nodes). Execution times are reported for the main steps of the pipeline analysis. From the Table it is evident that the execution time of the pipeline is strongly dependent on mpi-BLAST analyses. Therefore the behavior of the pipeline in terms of scalability and execution times is strongly influenced by BLAST comparisons (on single EST and on contigs).

**Figure 2:**
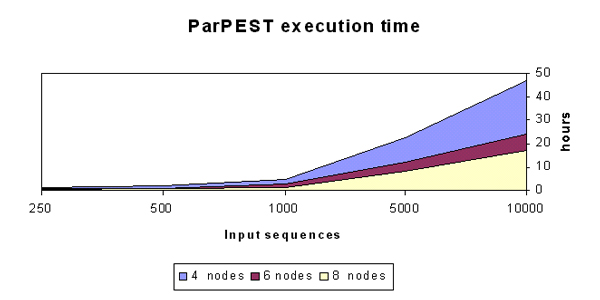
**Global execution time of PARPEST**. Results are shown to compare execution time of the pipeline with different number of working nodes. Time is reported in hours.

**Figure 3:**
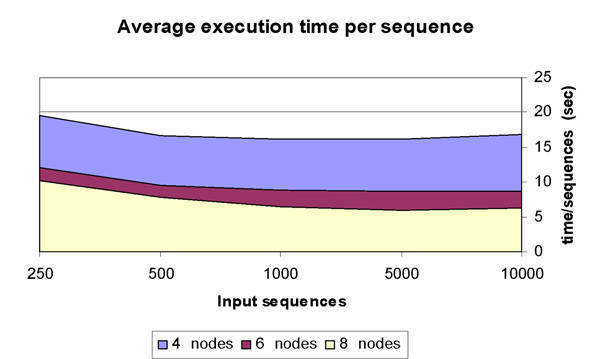
**Average execution time per sequence**. Data are reported to compare the average execution time per sequence in datasets of different dimension using a different number of working nodes. Time is reported in seconds.

As expected, large datasets (>1000 ESTs) give the widest reduction of execution time increasing the number of nodes (Figure 2). The execution time for smaller datasets is almost the same when using different node configurations. This is due to the overhead time caused by the job scheduling software. A deeper evaluation of the overhead time effect is reported in figure 3, which shows the average execution time per sequence using different node configurations. For increasing numbers of ESTs the profiles in figure 3 become flatter, because the average system response time becomes more stable for large data amounts, resulting in a reduced overhead effect.

We implemented the software to make it independent of the resource manager server. Therefore, though we based the system on a PBS resource manager, it can be easily ported to other environment such as Globus Toolkit [33] or SUN Grid Engine (SGE) [34]. Therefore the current pipeline could be also implemented on to the latest GRID computing environment.

### Database description

**Figure 4:**
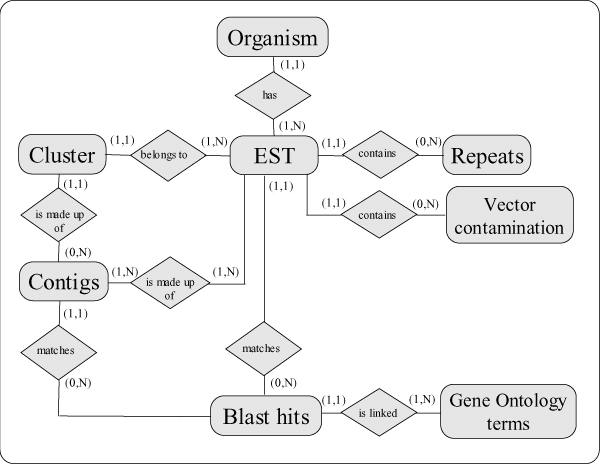
**The Entity-Relationship (ER) diagram of the MySQL database**. The ER diagram is reported to show the database structure schema. The schema describes the entities included in the database and their relationships.

dbEST data are organized in GenBank format where organism, cloning library, development stage, tissue specificity and other information are usually available. While parsing the input file, a complete set of basic information useful to described the sequence are collected in the 'est' table of the relational database we designed (Figure 4).

Another table is used to describe vector contaminations according to the report the main process of the pipeline automatically produces during the pre-processing step (Figure 1). Therefore the database will include information about the ESTs still including vector or linker contaminating sequences. A similar approach is used to report masked regions representing low complexity subsequences or repeats as identified byRepeatMasker, using RepBase as the filtering database.

Clusters obtained from PaCE resulting in single EST sequence or in contigs, are collected in the database too. A specific routine included in the main process of the pipeline performs a deep analyses on the clustered sequence to derive information on how many ESTs belong to a single contig, and how many contigs are produced once the sequences are clustered.

CAP3 assemblies sequences building a multiple alignment and deriving a consensus to obtain a contig. To use only high-quality reads during assembly, CAP3 removes automatically 5' and 3' low-quality regions (clipping step). Therefore, to keep information about the whole assembly process, both the complete alignment and the EST trimmed regions are recorded into the database.

The table designed to organize the BLAST report from raw EST data as well as from contig sequence analyses, can include the five most similar subject sequences and their related information. Gene Ontology terms related to each BLAST hit are recorded into the GO table included in the database.

### Web application

**Figure 5:**
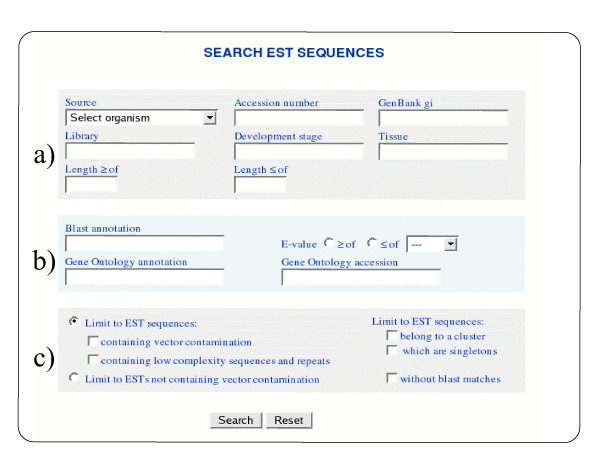
**A screenshot of the EST Browser**. ESTs can be retrieved by sequence features collected in the input step (a); by functional annotations (b); by specific properties reported by their processing.

The information obtained from the execution of the pipeline is stored in a MySQL database that provides a datawarehouse useful for further investigations. Indeed, all the information collected in the database can support biologically interesting analyses both to check the quality of the experimental results and to define structural and functional features of the data. For this purpose the database can be queried through SQL calls implemented in a suitable PHP-based interface. We provide a pre-defined web based query system to support also non expert users. Different views are possible. In particular, EST Browser (Figure 5) allows users to formulate flexible queries considering three different aspects, related to the features of the EST dataset as they have been described in the input process (Figure 5a). Therefore, a single EST or a group of ESTs can be selected by organism, clone library, tissue specificity and/or developmental stage. Searches can be filtered according to sequence lengths too.

Users can further select data based on the preliminary functional annotation, specifying a biological function as well as a GO term or a GO accession (Figure 5b). Moreover, restrictions on results obtained from the whole analytical procedure can be applied to retrieve different sets of ESTs (Figure 5c). For example users can retrieve all ESTs containing or not vectors, presenting or not BLAST matches, classified as singletons or to be in a cluster.

**Figure 6:**
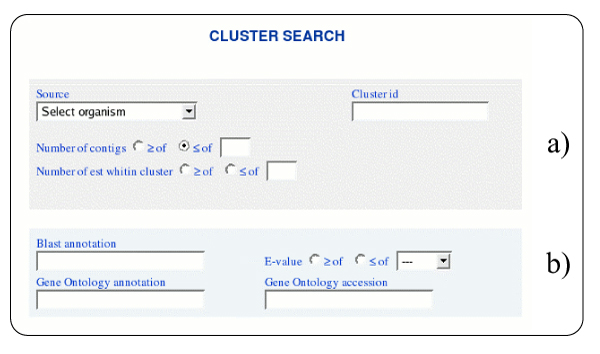
**A screenshot of the CLUSTER Browser**. Clusters can be retrieved using their identifier in the database and their structural features (a); functional annotation can be included in the query (b).

**Figure 7:**
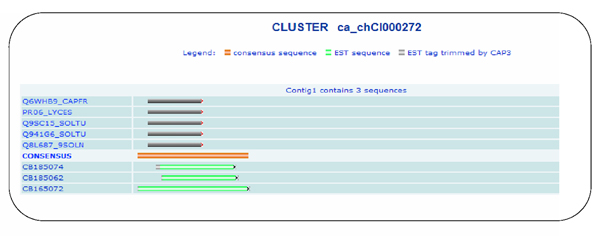
**A screenshot of the graphical organization of a cluster**. Windows reporting the length of the assembled contig (line in orange), the alignment of the ESTs that build the contig (in green) and the regions of the contig involved in blast matches (in dark grey). In light grey trimmed regions are reported too.

Cluster Browser (Figure 6) is specifically dedicated to select clustered sequences through a specific identifier, as it is assigned by the software, and their structural features (Figure 6a). Information about the functional annotation of the contig can be used for retrieving too (Figure 6b). Results from specific queries are reported in graphical display, reporting among other information, the contig sequence, the ESTs which define the clusters and their organization as aligned by CAP3 (Figure 7). This is considered useful to support analyses of transcribed variants putatively derived from the same gene or from gene families.

## Conclusion

We designed the presented pipeline to perform an exhaustive analysis on EST datasets. Moreover, we implemented ParPEST to reduce execution time of the different steps required for a complete analysis by means of distributed processing and of parallelized software. Though some efforts are reported in the literature where all the steps included in a EST comprehensive analyses are integrated in a pipelined approach [11-13], to our knowledge, no public available software is based on parallel computing for the whole data processing. The time efficiency is very important if we consider that EST data are in continuous upgrading.

The pipeline is conceived to run on low requiring hardware components, to fulfill increasing demand, typical of the data used, and scalability at affordable costs.

Our efforts has been focused to fulfill all the possible automatic analyses useful to highlight structural features of the data and to link the resulting data to biological processes with standardized annotation such as Gene Ontology and KEGG. This is fundamental to contribute to the comprehension of transcriptional and post-transcriptional mechanisms and to derive patterns of expression, to characterize properties and relationships and uncover still unknown biological functionalities.

Our goal was to set up an integrated computational platform, exploiting efficient computing, including a comprehensive informative system and ensuring flexible queries on varied fundamental aspects, also based on suitable graphical views of the results, to support exhaustive and faster investigations on challenging biological data collections.

## Availability

The design of the platform is conceived to provide the pipeline and its results using a user friendly web interface. Upon request, users can upload GenBank or Fasta formatted files.

We offer free support for processing sequence collections to the academic community under specific agreements. We would welcome you to find contacts and to visit a demo version of the web interface at .

## Figures and Tables

**Table 1:** Execution times for different node and data-set configurations. The execution times (in seconds) are collected for each step of the pipeline: 1) Blast on ESTs: functional annotation of raw EST sequences; 2) Pre-processing: vector contaminations cleaning and low complexity and interspersed repeat sequences masking; 3) Clustering; 4) Assembling; 5) Blast on Contigs: functional annotation of consensus sequences. Tot: is the global execution time of the pipeline.

	**#sequences**	**Blast on ESTs**	**Pre-processing**	**Clustering**	**Assembling**	**Blast on Contigs**	**TOT**
**4 nodes**	250	3712	441	15	201	501	4870
	500	7072	613	15	201	441	8342
	1000	13643	857	30	202	1474	16206
	5000	70490	2979	150	257	6806	80682
	10000	14559	6029	346	328	16045	168287

**6 nodes**	250	1992	441	15	201	350	2999
	500	3648	443	15	201	421	4728
	1000	6911	847	30	212	903	8903
	5000	35647	2834	136	268	4137	43022
	10000	72525	5483	240	357	7845	86450

**8 nodes**	250	1600	441	15	201	280	2537
	500	2517	443	15	202	461	3910
	1000	4704	797	30	212	733	6476
	5000	23819	2784	121	267	2853	29844
	10000	48700	5377	240	357	7845	62519
